# Effectiveness, durability and safety of dolutegravir and lamivudine versus bictegravir, emtricitabine and tenofovir alafenamide in a real-world cohort of HIV-infected adults

**DOI:** 10.1371/journal.pone.0291480

**Published:** 2023-09-29

**Authors:** Inés Mendoza, Alicia Lázaro, Alfredo Espinosa, Lorenzo Sánchez, Ana María Horta, Miguel Torralba

**Affiliations:** 1 Pharmacy Department, Hospital Universitario de Guadalajara, Guadalajara, Spain; 2 Alcalá University, Alcalá de Henares, Spain; 3 Internal Medicine Department, Research Unit, Hospital Universitario de Guadalajara, Guadalajara, Spain; University "Magna Graecia" of Catanzaro, ITALY

## Abstract

**Objective:**

Dolutegravir plus lamivudine (2-DR) is suggested as an initial and switch option in HIV-1 treatment. The aim of this study was to analyze the effectiveness, durability, and safety of 2-DR compared to bictegravir/emtricitabine/tenofovir alafenamide (3-DR).

**Patients and methods:**

This was an observational, ambispective study that included all treatment-naïve (TN) and treatment-experienced (TE) people living with HIV/AIDS (PLWH), who started 2-DR or 3-DR between 01 July 2018, and 31 January 2022. The primary endpoint was non-inferiority, at 24 and 48 weeks, of 2-DR vs 3-DR regarding the percentage of PLWH with viral load (VL)<50 and 200 copies/mL in TN (12% margin) and VL≥50 and 200 copies/mL in TE (4% margin). Durability of response and safety were also measured.

**Results:**

292 PLWH were included (39 TN and 253 TE). In TN PLWH, non-inferiority was not achieved at 24 weeks (17; 95% CI -17 to 51 p = 0.348). By week 48, all PLWH on 3-DR maintained VL<50 copies/mL compared to 70% of PLWH on 2-DR although without reaching statistical significance (-33; 95% CI -60 to -10 p = 0.289). Non-inferiority was not achieved in TE PLWH either at 24 (0.4; 95% CI -9 to 10 p = 1) or at 48 weeks (4.5; 95% CI -0.5 to 9 p = 0.132). In TN, the risk of treatment discontinuation was similar between groups (HR: 0.31, p = 0.07); similar rates were also found in TE (HR: 1.3, p = 0.38). TE PLWH on 2-DR showed a better safety profile compared to 3-DR (p = 0.017).

**Conclusion:**

Our results did not show non-inferiority in terms of virological effectiveness. Additionally, durability and safety of 2-DR were confirmed to be similar to 3-DR.

## Introduction

The introduction of antiretroviral therapy (ART) has considerably improved the life expectancy of people living with HIV/AIDS (PLWH) [1]. As a result of this improvement in morbidity and mortality, HIV is now a chronic disease. Although contemporary ART regimens have a better safety profile compared to early ART, they have been associated with long-term toxicity [2, 3].

Historically, the use of nucleoside reverse transcriptase inhibitors (NRTIs) as monotherapy or dual therapies rapidly led to treatment failure due to the selection of resistance mutations [4]. However, two-drug ART regimens (2-DR) have been studied to minimize long-term drug exposure, reduce drug-drug interactions (especially in polymedicated PLWH), reduce costs, and facilitate adherence [2, 5, 6].

Since 2019, HIV treatment guidelines have recommended three-drug regimens with bictegravir/emtricitabine/tenofovir alafenamide (BIC/FTC/TAF) and dolutegravir/lamivudine/abacavir, and a two-drug regimen with dolutegravir/lamivudine (DTG/3TC), all containing integrase strand transfer inhibitors (INSTIs), as approved combinations for initial treatments [7–9].

Simplification strategies with 2-DRs like DTG/3TC have demonstrated their effectiveness in clinical trials in treatment-naïve (TN) and treatment-experienced (TE) adults [10, 11] with viral load (VL) <500 000 copies/mL [12]. Real-world data has also shown the effectiveness and safety of 2-DRs with DTG/3TC in naïve PLWH [13]. To our knowledge, there are no studies comparing 2-DRs with DTG/3TC versus the 3-DR standard of care, BIC/FTC/TAF [9, 14].

Additionally, the generalizability of clinical trials results is poor because they are performed in conditions very different from real-life usual care. Real-world studies on effectiveness, durability and safety of 2-DRs compared to 3-DR standard of care are needed to confirm clinical trial results and support their use in clinical practice.

To assess the non-inferiority of a 2-DR with DTG/3TC versus a 3-DR with BIC/FTC/TAF, and to compare the immunological effectiveness, durability and safety in an intention-to-treat (ITT)-exposed analysis in a real-world cohort of HIV-1 TN and TE PLWH.

## Materials and methods

### Design

We conducted an ambispective observational study of HIV-1-infected TN and TE PLWH treated with 2-DR (DT/3TC) or 3-DR (BIC/FTC/TAF) in a healthcare area of Spain between 01 July 2018 and 31 January 2022. The study protocol was approved in 16^th^ of September of 2020 by the Institutional Review Board of the hospital (Comité de Ética de la investigación con Medicamento del Área de Salud de Guadalajara) and registered with the Spanish Agency for Medicines and Medical Devices (AEMPS). The approval number is ESPA DTG/3TC.

### Participants and setting

The study sample included all TN and TE PLWH who initiated treatment with a 3-DR (BIC/FTC/TAF) or 2-DR (DTG/3TC). TN PLWH were defined as people with no previous exposure to ART; TE who had discontinued ART and presented VL >1000 copies/mL were also considered TN. Exclusion criteria were: pregnancy, documented or suspected resistance to 3TC or DTG, hepatitis B virus infection (for DTG/3TC), and a history of adverse events (AEs) to either drug; those who initiated study treatment or were followed up in another hospital or PLWH participating in clinical trials were also excluded.

PLWH were followed up from the date of ART initiation to the first date of treatment discontinuation (stopping or switching therapy) or censoring.

### Interventions

All data were collected from the patient’s electronic medical record and drug prescription program. Demographic and baseline disease characteristics included: age, sex, hepatitis B virus infection (HBV), hepatitis C virus infection (HCV), time since HIV diagnosis, time since first ART, number and type of previous ART, mean nadir CD4+ lymphocyte cell count at any time and pre-treatment CD4+ cell count. Log_10_ VL was measured in TN PLWH, while the percentage of PLWH with VL <50 and VL <200 copies/mL was measured in TE.

VL was measured at the time closest to the 24-48-week point (the lowest period between two measures was 16 weeks) according to clinical practice using the Roche Molecular Cobas Ampliprep Taqman HIV v2.0 (limit of detection: 20 HIV-1 RNA copies/mL). Virological failure (VF) was defined as two consecutive HIV-1 RNA ≥50 copies/mL (with an interval of 1–2 months) [15]. A genotypic resistance test was performed if VL was >1000 copies/mL, in accordance with clinical practice. Immunological effectiveness was measured as an increase in the CD4+ count 24 and 48 weeks after starting treatment.

Additionally, time to treatment discontinuation and all-cause discontinuation was assessed among all PLWH and classified into AE, VF, lost to follow-up, death, treatment simplification, interactions, pregnancy and treatment non-adherence. PLWH were considered as lost to follow-up if they had no contact with the clinician or pharmacist for more than 6 months (the usual period between each clinical visit at the hospital). We analyzed possible risk factors associated with treatment discontinuation. The incidence of AEs was assessed at each hospital visit by clinicians and pharmacists through medical records.

### Outcome

The primary study endpoint was to assess the non-inferiority in terms of virological effectiveness of 2-DR versus 3-DR in TN and TE PLWH at 24 and 48 weeks. In TN, virological effectiveness was measured as the proportion of PLWH with VL <50 copies/mL and VL <200 copies/mL with a non-inferiority margin of 12%, while in TE, effectiveness was defined as the proportion of PLWH with virological failure (VF) (VL ≥50 copies/mL and VL ≥200 copies/mL) with a non-inferiority margin of 4%. The incidence of VF, resistance mutations and median VL in PLWH with VF were also assessed. All analyses were carried out in an ITT-exposed population, which included all PLWH who received at least one dose of 2-DR or 3-DR. Secondary endpoints included changes in the CD4+ count from baseline at 24 and 48 weeks, treatment durability, risks and reasons for discontinuation, and safety (rate and type of AE and frequency of treatment discontinuation due to AE).

### Statistical analysis

Descriptive statistics were used to evaluate initial PLWH data including clinical, laboratory and demographic characteristics, and primary and secondary outcomes. Results are presented as median and interquartile ranges, as appropriate. Mean and standard deviation were calculated for quantitative variables, and the Student t-test was used to compare groups. The chi-square test was applied for analyzing differences in qualitative values, as appropriate. Absolute and relative frequencies were calculated for qualitative variables followed by the application of the Pearson chi-square test or Fisher chi-square test if necessary. Treatment durability was analyzed using Kaplan-Meier survival analysis and Cox regression with 95% confidence limits (95% CI). Statistical analyses were performed with Stata 15.0. We assessed non-inferiority with a conventional 95% CI approach for the difference in the proportion of PLWH with a VR (3-DR group minus 2-DR group) with a prespecified non-inferiority margin of −12% in TN group and 4% in TE group based on published US FDA regulatory guidance [16].

## Results

### Number of participants

**Fig 1:**
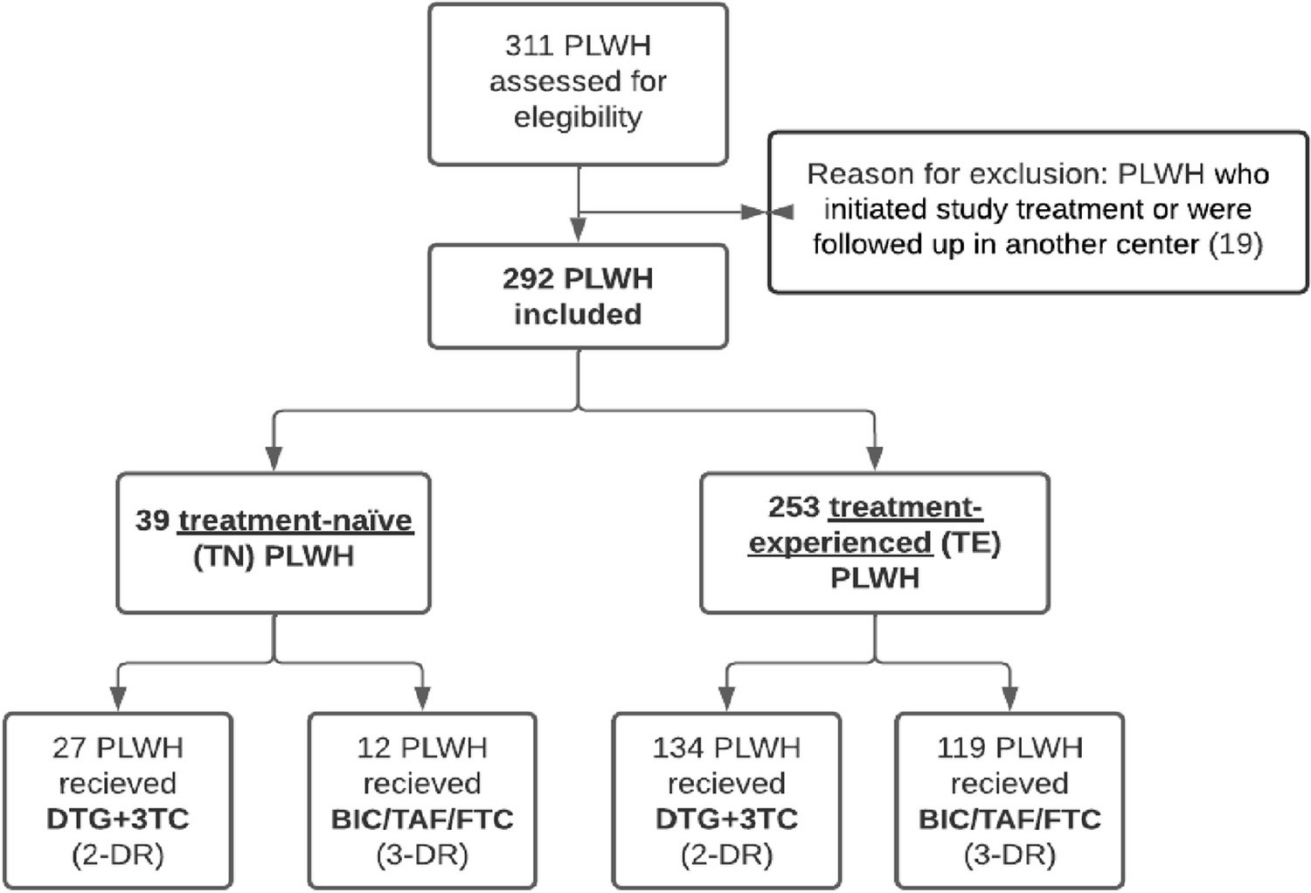
Flow-chart of treatment-naïve and treatment-experienced patients. Abbreviations: 3TC, lamivudine; BIC/FTC/TAF: bictegravir/emtricitabine/tenofovir alafenamide; DTG: dolutegravir; PLWH: people living with HIV/AIDS.

A total of 311 PLWH were initially identified. Nineteen PLWH started treatment in other centres, and so were excluded from the analysis, leaving 292. The percentage of TN PLWH was 13.4% (n = 39), of whom 69% (n = 27) were on 2-DR. In the TE group, 53% (n = 134) were on 2-DR (Fig 1). The median follow-up in TN PLWH was 54 (28–102) weeks for PLWH on 2-DR and 26 (9–57) weeks for those on 3-DR. In the TE group, the median follow-up was 63 (37–140) weeks in 2-DR PLWH and 57 (28–98) weeks in 3-DR. PLWH in the 3-DR group were more frequently coinfected with HBV and HCV compared with those in the 2-DR group. The characteristics of the study population are summarized in Table 1.

**Table 1:** Demographic and baseline characteristics of treatment-naïve and treatment-experienced PLWH with two- and three-drug regimens.

Baseline Parameter	Treatment-naïve			Treatment-experienced PLWH		
	PLWH					
	Two-drug	Three-drug	p	Two-drug	Three-drug	p
	(2-DR)	(3-DR)		(2-DR)	(3-DR)	
	(n = 27)	(n = 12)		(n = 134)	(n = 119)	
Age (years)^a^	35 (28–50)	40 (25–47)	0.29	48 (40–54)	49 (40–57)	0.39
Male, n (%)	20 (74)	11 (92)	0.39	91 (68)	86 (72)	0.39
HCV coinfection, n (%)^b^	0	1 (8.3)	0.3	2 (1.5)	3 (2.5)	0.67
HBV coinfection, n (%)^c^	0	2 (17)	0.09	0	7 (6)	**0.004**
Time from diagnosis of HIV infection ^a^^,^^d^	18 (8–28)	5 (4–22)	0.23	9.8 (4–20)	15 (7–25)	**0.03**
Time from first HIV treatment (years) ^a^	NA	NA	NA	7 (3–14)	8 (3–15)	0.32
Log VL at diagnosis ^a^	4.5 (4.1–5)	4.8 (3.7–5.5)	0.47	4.9 (4.3–5.4)	5.1 (4.7–5.6)	0.41
Log VL at study treatment initiation ^a^	4.1 (3.8–4.7)	4.6 (3.5–5.4)	0.2	NA	NA	NA
VL<50 copies/mL at study treatment initiation ^a^, n (%)	NA	NA	NA	115 (86)	93 (78)	0.14
VL<200 copies/mL at study treatment initiation ^a^, n (%)	NA	NA	NA	127 (95)	105 (88)	0.06
CD4+ T-cell count nadir (cells/μL) ^a^	360 (265–585)	360 (30–530)	0.65	215 (78–363)	223 (80–310)	0.15
CD4+ T-cell count (cells/μL) at study treatment initiation ^a^	460 (330–590)	335 (35–527)	0.25	720 (470–920)	700 (440–928)	0.92
Regimen at switching mean of ART (SD)	NA	NA	NA	2.5 (1.5)	3.1 (2)	**0.02**
NRTI+ NNRTI, n (%)	NA	NA	NA	29 (22)	14 (12)	**0.04**
NRTI+ INSTI, n (%)	NA	NA	NA	86 (64)	84 (72)	0.2
NRTI+ PI, n (%)	NA	NA	NA	8 (6)	6 (5)	0.77
Others, n (%)	NA	NA	NA	2 (1.5)	2 (1.7)	0.46

Abbreviations: ART, antiretroviral therapy; HCV, hepatitis C virus; INSTI, integrase strand transfer inhibitors; NA, not applicable; NNRTI, non-nucleoside reverse-transcriptase inhibitor; NRTI, nucleoside reverse-transcriptase inhibitor; PI, protease inhibitor; VL, viral load.

^a^Data are presented as median (p25-p75).

^b^Positive HCV antibodies and positive HCV-RNA

^c^Positive HBsAg.

^d^ Time is presented in days in treatment-naïve PLWH and in years in treatment-experienced PLWH.

With respect to baseline characteristics, time since diagnosis of HIV infection (years) (p = 0.03) and the number of previous ART (p = 0.02) were significantly higher in 3-DR PLWH in the TE group; more PLWH in the 2-DR group had previously received 2 NRTIs + non-nucleoside reverse transcriptase inhibitors (NNRTI) (22%) compared to 3-DR PLWH (12%) (p = 0.04). The rest of the baseline characteristics were statistically similar.

### Virological effectiveness

Most TN PLWH on 2-DR achieved a virological response (VR): 84% (VL <50 copies/mL) and 92% (VL <200 copies/mL) within 24 weeks of initiating 2-DR versus 67% and 100%, respectively, for PLWH on 3-DR. Non-inferiority was not achieved at 24 weeks (17; 95% CI -17 to 51 p = 0.348) (VL <50 copies/mL). By week 48, all PLWH on 3-DR maintained VL<50 copies/mL compared to 70% of PLWH on 2-DR, although without reaching statistical significance (-33; 95% CI -60 to -10 p = 0.289). However, considering a VL of <200 copies/mL, 90% of the 2-DR group presented VL<200 copies/mL versus 100% of PLWH on the 3-DR (-10; 95% CI -23 to -3 p = 1).

Regarding TE PLWH, 13% presented VL ≥50 copies/mL within 24 weeks of initiating 2-DR, and 5.6% VL ≥200 copies/mL; compared to those of TE on 3-DR, 12% and 1.4%, respectively. Non-inferiority was not achieved in TE PLWH either at 24 (0.4; 95% CI -9 to 10 p = 1) or at 48 weeks (4.5; 95% CI -0.5 to 9 p = 0.132).

**Fig 2:**
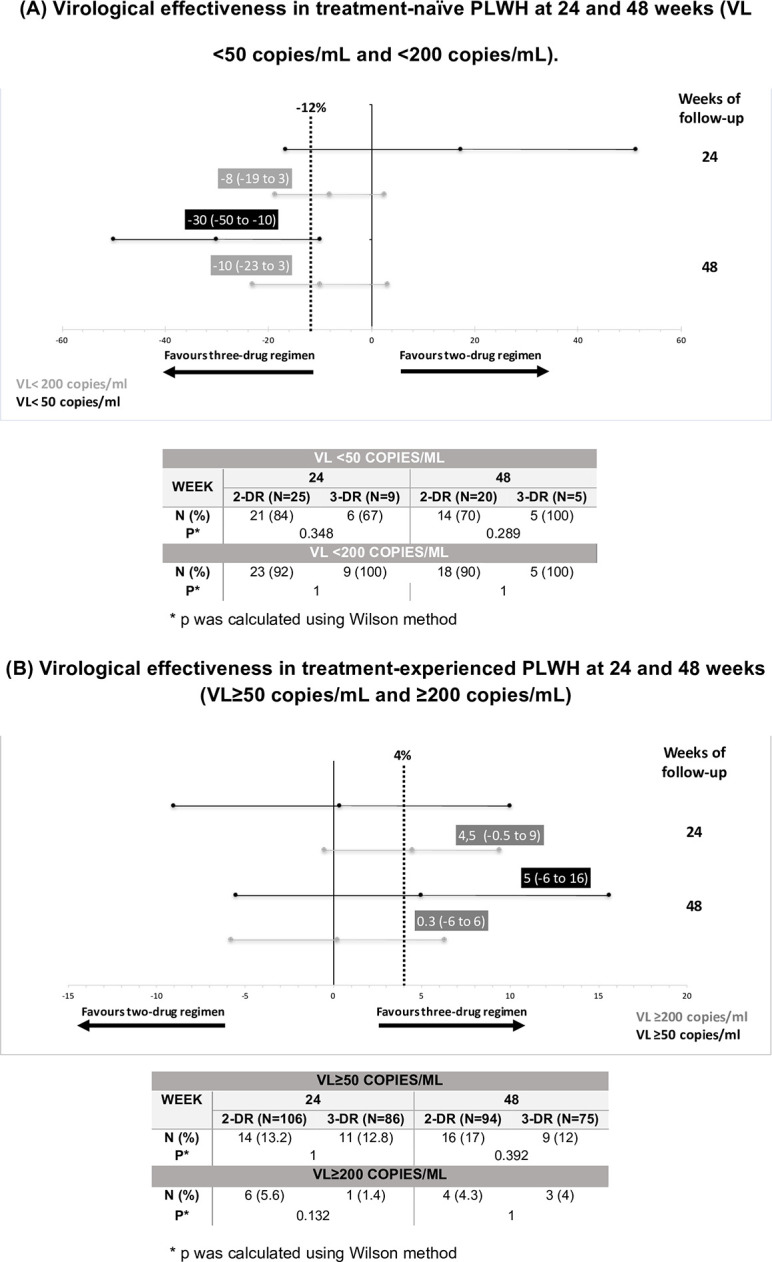
Forest plot of virological effectiveness of 2-DR compared to 3-DR in treatment-naïve (A) and treatment-experienced (B) PLWH analyzed separately. In naïve PLWH (A), non-inferiority in terms of effectiveness was expressed as the difference between the proportion of 2-DR and 3-DR PLWH with VL <50 copies/mL (black) and <200 copies/mL (gray) at 24 and 48 weeks. In treatment-experienced PLWH (B), non-inferiority in terms of effectiveness was expressed as the difference between the proportion of 2-DR and 3-DR PLWH who achieved V1_50 copies/mL and >200 copies/mL at 24 and 48 weeks.

Treatment differences between the proportion of 2-DR and 3-DR TN PLWH with VL <50 copies/mL and <200 copies/mL, and the difference in the proportion of TE PLWH who reached VL ≥50 copies/mL and ≥200 copies/mL at weeks 24 and 48 are detailed in Fig 2A and 2B, respectively.

### Virological failure

TN and TE PLWH on 2-DR and 3-DR did not show differences in VF considering a VL of ≥50 copies/mL or VL of ≥200 copies/mL. Among the 39 TN in the analysis, four PLWH in the 2-DR group presented VF considering a VL of ≥50 copies/mL, but only two PLWH had VF considering a VL of ≥200 copies/mL. The median VL in 2-DR PLWH was 191 copies/mL (94 to 15297). Only two PLWH presented VF in the 3-DR group, with a median VL of 250 copies/mL. No TN PLWH discontinued treatment because of VF, and no resistance mutations were reported in this group.

In the TE group, 11.2% of PLWH on 2-DR had VF considering a VL ≥50 copies/mL and 4.5% considering a VL ≥200 copies/mL. The median VL in this group was 109 copies/mL (85 to 1163). Two PLWH discontinued treatment because of VF. A resistance mutation was confirmed in one PLWH (*M184I*). In the 3-DR group, 7.6% of PLWH presented VF (p = 0.33), and 1.7% had a VL≥ 200 copies/mL, with a median VL of 182 copies/mL (83 to 244) (p = 0.29).

### Immunological effectiveness

In TN PLWH, increases in the CD4+ count from baseline were similar between the 2-DR and 3-DR groups at 24 weeks: 207 cells/μL in 2-DR and 376 cells/μL in 3-DR (p = 0.08). Increases were also comparable at 48 weeks: 242 cells/μL and 274 cells/μL (p = 0.71) for 2-DR and 3-DR PLWH, respectively.

With regard to TE PLWH, no statistically significant changes were found in the number of CD4+ cells/μL. The CD4+ count increased by 14 cells/μL in 2-DR PLWH and by 47 cells/μL in 3-DR PLWH (p = 0.34) at week 24. Similarly, at 48 weeks, the count increased by 34 cells/μL and decreased by 1.2 cells/μL, respectively (p = 0.32).

### Durability of treatment

**Fig 3:**
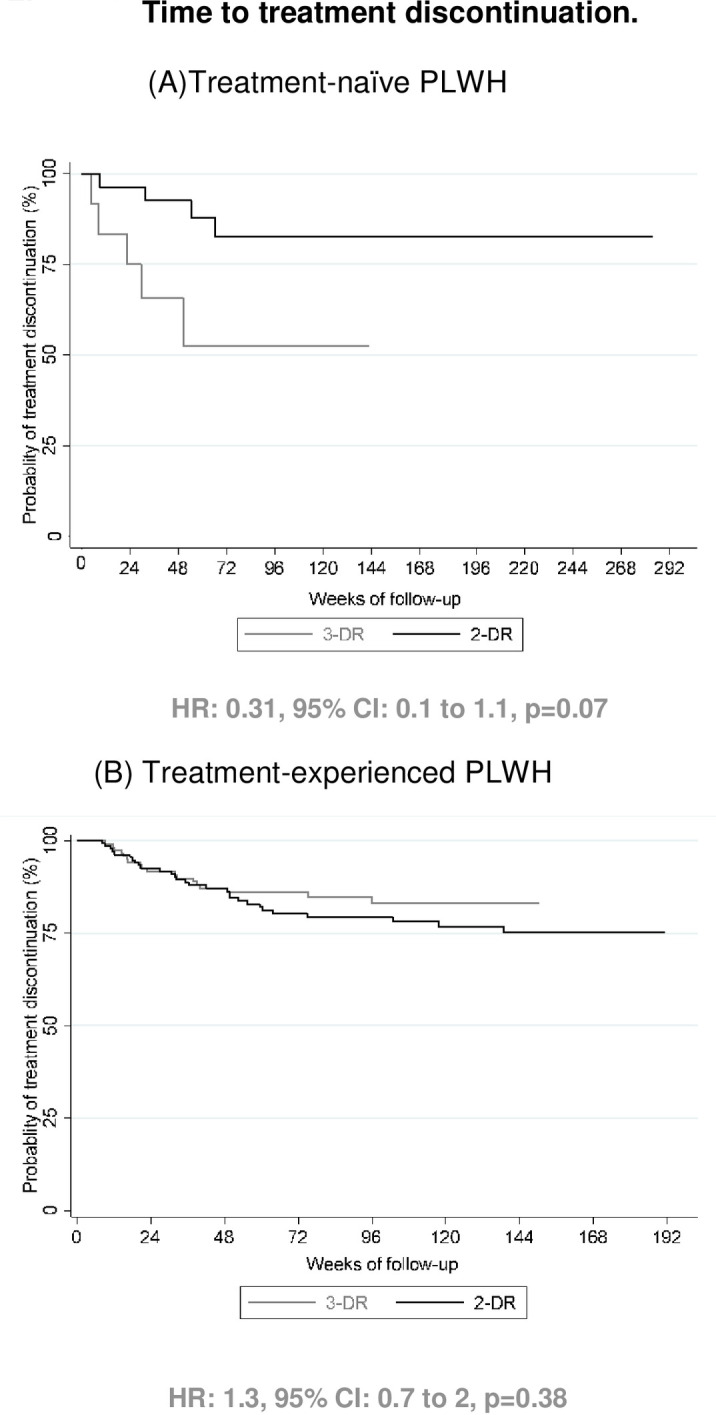
Kaplan-Meier analysis of time from 2-DR (grey) and 3-DR (black) initiation to discontinuation in treatment-naïve (A) and treatment-experienced (B) patients. The Hazard ratio (HR) is shown. Durability of treatment (interval between study treatment initiation and discontinuation) was calculated from the date of prescription to the doctor’s indication to stop treatment for any reason. Discontinuation due to treatment simplification was not included in the analysis.

The risk of treatment discontinuation in TN PLWH was similar in 2-DR and 3-DR groups (hazard ratio [HR]: 0.31, 95% CI: 0.1 to 1.1, p = 0.07). Similarly, no statistically significant differences were found in TE PLWH (HR: 1.3, 95% CI: 0.7 to 2, p = 0.38). The durability of treatment is expressed using Kaplan-Meier curves in Fig 3.

Reasons for treatment discontinuation varied among groups. Only five TN PLWH discontinued 2-DR: due to AEs (2) and lost to follow-up (3). Five PLWH in the 3-DR group also discontinued treatment: lost to follow-up (1), treatment simplification (1), death due to a progressive multifocal leukoencephalopathy (1), interactions (1) and pregnancy (1).

In the TE group, 32 PLWH (24%) discontinued 2-DR: lost to follow-up (16), VF (2), AEs (5), death not related to ART (non-small cell lung cancer, suicide and upper gastrointestinal bleeding) (3), treatment non-compliance (2), pregnancy (2) and participation in a clinical trial (2). Among the 3-DR group, 21 PLWH (18%) discontinued treatment: lost to follow-up (11), AE (6), death due to non-small cell lung cancer (1), and pregnancy (3).

By multivariate analysis, in TN PLWH, 2-DR had a lower risk of discontinued treatment (hazard ratio [HR]: 0.23, 95% CI: 0.05 to 1.1, p = 0.07), adjusted by age, sex, rate of PLWH with HCV coinfection, CD4+ T-cell count (cells/μL) and Log VL at study treatment initiation. In the same case, in TE PLWH, 2-DR presented a lower risk of treatment discontinuation (hazard ratio [HR]: 0.74, 95% CI: 0.3 to 2.1, p = 0.56), adjusted by age, sex, rate of PLWH with HCV coinfection, CD4+ T-cell count (cells/μL) at study treatment initiation, time from diagnosis of HIV infection and years from first HIV treatment, rate of PLWH with VL<200 copies/mL at study treatment initiation, and number of previous regimen of ART.

### Safety

Most AEs were reported as mild or moderate in severity. Five TN PLWH on 2-DR (19%) reported AEs; the median number of AEs per PLWH was two (IQR: 1–2.5), and they occurred in only one PLWH in the 3-DR group (8%). The groups did not differ significantly in the rate of AEs (p = 0.65).

Regarding the TE group, 12 PLWH on 2-DR (9%) had at least one mild/moderate AE compared to 23 PLWH on 3-DR (19%), (p = 0.017). The median number of AEs per PLWH was 1 (IQR: 1 to 1) and 1 (IQR: 1 to 2), respectively.

**Fig 4:**
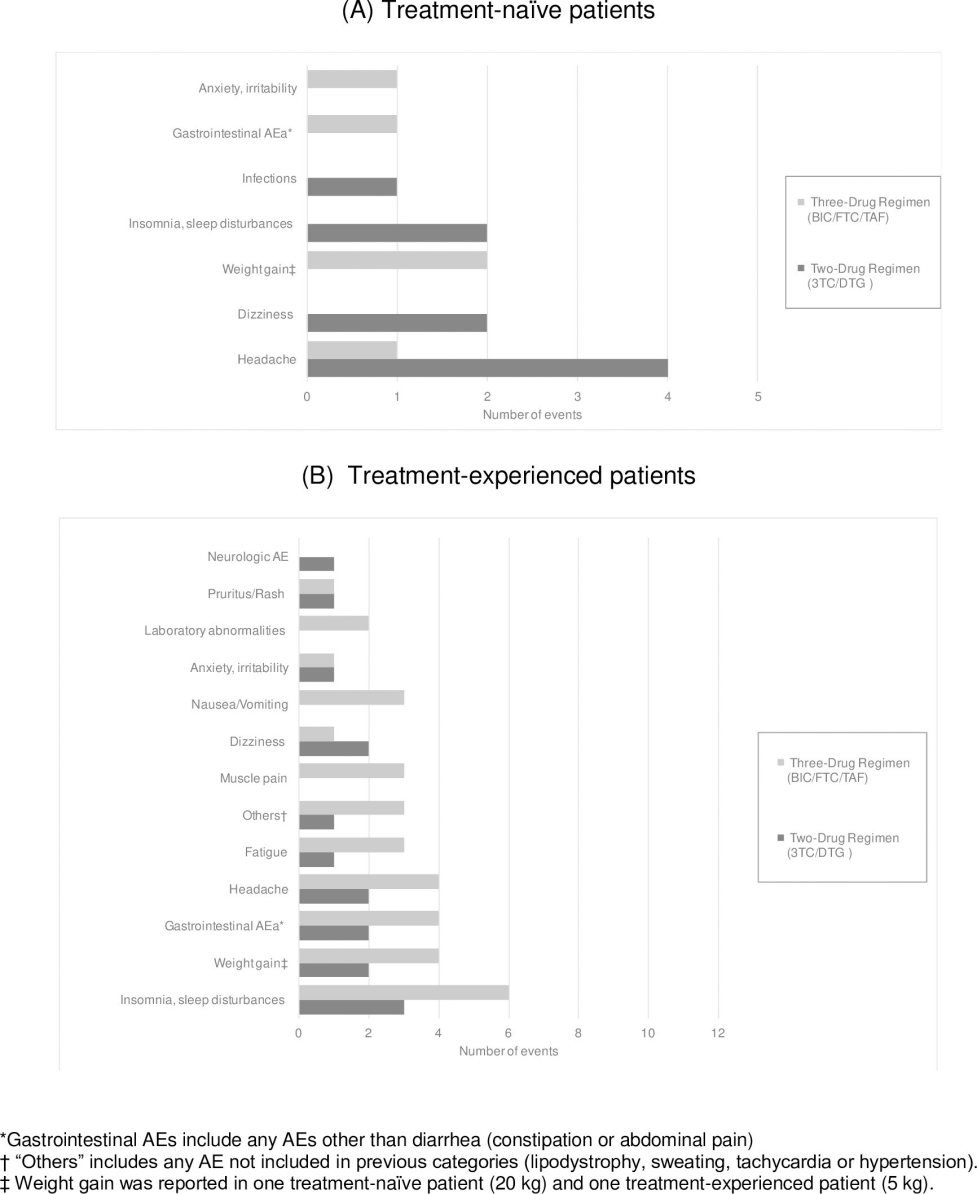
Most frequent adverse events reported in treatment-naïve (A) and treatment-experienced (B) patients comparing treatment groups: 2-DR (black) and 3-DR (grey).

The most frequently reported AEs in TN and TE per treatment group are shown in Fig 4.

No differences were found between AE-related discontinuation in TN PLWH. Two PLWH in the 2-DR group discontinued the study treatment compared to no PLWH in the 3-DR group. In the TE group, four PLWH discontinued 2-DR because of AEs and five in the 3-DR group. No statistical differences were found between groups in either TN (p = 1) or TE (p = 0.74).

## Discussion

Both bictegravir and dolutegravir are considered second-generation INSTIs, and are known to possess a similarly high genetic barrier. They are the standard-of-care treatment recommended for TN and TE PLWH in national and international guidelines [9, 12]. Although available 3-DR regimens are effective and generally safe, issues of toxicity and cost remain over the length of exposure [17]. DTG plus 3TC may be a useful option to address these issues [18]. This regimen has shown promising efficacy in previous clinical trials with TN [19] and TE PLWH [20] compared to DTG-based 3-DRs. However, comparisons to the standard of care, BIC/FTC/TAF, have yet to be performed.

Our results did not show non-inferiority in terms of virological effectiveness, either in TN or in TE PLWH.

Additionally, no statistically significant differences between groups were found regarding the risk of treatment in TN and TE PLWH. In terms of safety, 2-DR showed a better safety profile with fewer AEs per PLWH than the 3-DR in the TE group, and a similar safety profile in TN PLWH.

Most characteristics at baseline were well balanced. It should be noted that the 3-DR group were more treatment-experienced (higher number of previous ART) and had a longer time since diagnosis of HIV infection compared to 2-DR PLWH. This could be explained by the fact that most previous ART prescribed in 3-DR PLWH was elvitegravir/cobicistat/emtricitabine/tenofovir, a regimen commonly used in TE PLWH. However, 2-DR PLWH switched from dolutegravir/abacavir/lamivudine (DTG+ABC+3TC), which was commonly used in naïve PLWH according to national guidelines [21].

With respect to virological effectiveness in TN, PLWH on the 2-DR presented a higher VR by week 24 considering VL <50 copies/mL. However, all 3-DR TN achieved VR by week 24 (considering VL <200 copies/mL) and week 48, regardless of VL. It should be noted that all 3-DR PLWH reached VR at week 48 (considering VL ≤50 copies/ml) but this result was not significant. Although 3-DR presented a higher rate of VR compared to 2-DR, superiority was not an outcome of this study.

Rates of VF in TN PLWH on 2-DR were lower compared to other real-world studies with DTG/3TC at 24 weeks [22, 23] and 48 weeks [13]. This could be explained by the small number of TN PLWH who completed the 48 weeks of follow-up. However, rates of VF in TN PLWH on 3-DR were similar compared to other publications [24]. In the same way, the preliminary results of this study at 24 weeks concluded that the effectiveness of 2-DR was similar to 3-DR in TN and TE PLWH [25].

Regarding TE PLWH, non-inferiority was not demonstrated in any measurement. Rates of VF in TE PLWH with 2-DR were comparable to other studies in clinical practice [26].

No differences were found between 2-DR and 3-DR in terms of incidence of VF. Additionally, median VLs in PLWH who experienced VF were lower compared to other studies. Of note, a study that compared bictegravir- versus dolutegravir-based regimens established that the association between the choice of core agent and the occurrence of low-level viraemia remained statistically insignificant [27].

One TE PLWH in the 2-DR group presented a resistance mutation (*M184I*). This could be explained by the low rate of treatment compliance reported in this PLWH and the fact that she was taking 3TG+DTG (two pills). This is contrary to data from clinical trials, [20, 28] where no drug resistance mutation was found.

Considering effectiveness in terms of immunological response, the data described here suggest that both regimens have similar effectiveness in TN and TE PLWH, as the differences between the mean CD4+ cell counts in 2-DR and 3-DR were not statistically significant.

TN and TE PLWH presented a similar risk of discontinuation. This is consistent with previous reports in TE PLWH that compare BIC/FTC/TAF versus dolutegravir-containing regimens [29] or results of a meta-analysis of BIC/FTC/TAF compared to other antiretroviral regimens. A retrospective analysis of DTG +3TC versus BIC/FTC/TAF in virologically suppressed PLWH showed no significant differences between both groups of treatment. These results agree with our findings [30]. The main reason for discontinuation was loss to follow-up, mainly in the TE group. No association was found between baseline characteristics and the risk of treatment discontinuation.

Our data suggest similar tolerability of 2-DR and 3-DR in TN PLWH. However, rates of TN PLWH on 3-DR with any AE were fewer compared to a phase 3 study [29]. This could be explained by the closest monitoring point in the clinical trial compared to clinical practice.

Regarding the TE group, fewer 2-DR PLWH reported drug-related AEs than 3-DR PLWH, possibly because of the removal of abacavir (ABC) from the regimen DTG+ABC+3TC, to DTG/3TC and is consistent with the findings of a meta-analysis of 3-DR comparing it to other ART regimens, [31] although higher rates of PLWH with at least one mild/moderate AE were found in the 3-DR group compared to other studies [32]. This could be explained because most of the PLWH that started on 3DR received previously elvitegravir/cobicistat/emtricitabine/tenofovir alafenamide (one INSTI: "elvitegravir/c" is modified by another: "bictegravir"). Clinicians are more likely to report more AEs when ART is modified (switching to new drugs) than when a drug (ABC) is withdrawn from ART like ABC+3TC+DTG.

In contrast to clinical trials with a patient sample in a strictly standardized setting and rigorously controlled conditions that may compromise their external validity, [33] the moderate sample size of our study enabled us to provide key information in a real-world setting in a single healthcare area after inclusion of DTG/3TC and BIC/FTC/TAF in guidelines as standard-of-care treatment in TN and TE PLWH. The relatively long follow-up time and absence of exclusion criteria (e.g. VL>100 000 copies/mL, nadir CD4+ count <200 cells/μL, comorbidities, HCV coinfection or previous VF) may have had an impact on the effectiveness of the 2-DR in clinical practice. Unlike clinical trials that studied the efficacy of switching to DTG/3TC, our population mainly included PLWH with a long treatment history, providing a snapshot of ‘real-life’ data. Moreover, an important strength of this study is the inclusion of the newer standard-of-care according to guidelines as a comparator arm in order to assess effectiveness and safety in a real-world setting.

### Limitations

Our study is not without limitations. First, the follow-up time of PLWH on 2-DR was longer than those on 3-DR, both in TN and TE PLWH. This could diminish the reliability of the higher risk of treatment discontinuation in 3-DR TN PLWH.

Another study limitation is the smaller size of the 3-DR TN group. Nevertheless, it was also strengthened as the two groups were well-balanced for all variables, thus potentially limiting the confounders.

It should be noted that it has been difficult to compare our study to others because of the lack of studies that compare DTG/3TC and BIC/FTC/TAF. However, it offers an analysis of the real-life effectiveness of these two regimens when prescribed by HIV specialists, contributing real-world data.

Considering this, further studies are needed to properly assess the durability of this ART over time, especially in comparison with other strategies such as long-acting ART (cabotegravir plus rilpivirine).

## Conclusions

In summary, both optimization strategies have similar effectiveness profiles. However, non-inferiority criteria in terms of virological effectiveness were not met.

Additionally, DTG plus 3TC showed a high tolerability profile in TE PLWH, with long-term durability in TN PLWH.
